# LIFESPAN: A tool for the computer-aided design of longitudinal studies

**DOI:** 10.3389/fpsyg.2015.00272

**Published:** 2015-03-24

**Authors:** Andreas M. Brandmaier, Timo von Oertzen, Paolo Ghisletta, Christopher Hertzog, Ulman Lindenberger

**Affiliations:** ^1^Center for Lifespan Psychology, Max Planck Institute for Human DevelopmentBerlin, Germany; ^2^Department of Psychology, University of VirginiaCharlottesville, VA, USA; ^3^Faculty of Psychology and Educational Sciences, University of GenevaGeneva, Switzerland; ^4^Distance Learning University SwitzerlandBrig, Switzerland; ^5^Adult Cognition Lab, School of Psychology, Georgia Institute of TechnologyAtlanta, GA, USA; ^6^Max Planck University College London Centre for Computational Psychiatry and Ageing ResearchLondon, UK

**Keywords:** statistical power, structural equation modeling, latent growth curve modeling, optimal design, power equivalence theory, effective error

## Abstract

Researchers planning a longitudinal study typically search, more or less informally, a multivariate space of possible study designs that include dimensions such as the hypothesized true variance in change, indicator reliability, the number and spacing of measurement occasions, total study time, and sample size. The main search goal is to select a research design that best addresses the guiding questions and hypotheses of the planned study while heeding applicable external conditions and constraints, including time, money, feasibility, and ethical considerations. Because longitudinal study selection ultimately requires optimization under constraints, it is amenable to the general operating principles of optimization in computer-aided design. Based on power equivalence theory (MacCallum et al., 2010; von Oertzen, 2010), we propose a computational framework to promote more systematic searches within the study design space. Starting with an initial design, the proposed framework generates a set of alternative models with equal statistical power to detect hypothesized effects, and delineates trade-off relations among relevant parameters, such as total study time and the number of measurement occasions. We present LIFESPAN (Longitudinal Interactive Front End Study Planner), which implements this framework. LIFESPAN boosts the efficiency, breadth, and precision of the search for optimal longitudinal designs. Its initial version, which is freely available at http://www.brandmaier.de/lifespan, is geared toward the power to detect variance in change as specified in a linear latent growth curve model.

## Introduction

Describing, explaining, and modifying between-person differences in change are central goals in research on lifespan development (Baltes and Nesselroade, 1979; Hertzog, 1996; Baltes et al., 2006; Ferrer and McArdle, 2010; Lindenberger et al., 2011). Numerous studies have shown that people differ in rates of change in many functional domains, both at neural and behavioral levels of analysis (e.g., Lindenberger, 2014). To delineate the antecedents, correlates, and consequents of these differences, differences in change in variables of interest must be measured with sufficient reliability. Hence, researchers have begun to examine the relative importance of factors that contribute to the statistical power to detect between-person differences in change (represented by the variance in change), such as the true variance in change, the number and precision of indicators, the number and distribution of measurement occasions, and the total time elapsing from the beginning to the end of the study (henceforth referred to as total study time; Hertzog et al., 2006; von Oertzen et al., 2010; von Oertzen and Brandmaier, 2013; Rast and Hofer, 2014). The search for optimally powerful longitudinal research designs requires close and simultaneous attention to the relative contributions of each of these factors to statistical power.

There is a dire need for a coherent and unified approach to the *a*-*priori* estimation of statistical power that can efficiently assist researchers in identifying longitudinal research designs with optimal statistical power to detect key effects under a given set of assumptions and design constraints (Maxwell et al., 2008; Moerbeek, 2011). Current statistical power analysis is often based on Monte Carlo simulations (e.g., Hertzog et al., 2008; Ke and Wang, 2014; Rast and Hofer, 2014), which can be carried out with the help of statistical software packages such as M*plus* (Muthén and Muthén, 2007). However, the Monte Carlo simulation approach can be cumbersome, and requires scientists to choose how and when to simulate possible design configurations. What is currently needed is a method for an efficient yet comprehensive overview of the ways in which different parameter values or design configurations contribute to statistical power. Currently available dedicated software can be used for the *a*-*priori* power analysis of hypotheses about repeated measures means and interactions in a general linear model context (G^*^power; Faul et al., 2007) and for group differences in mean growth curve parameters, as in intervention contexts (Hedeker et al., 1999; Kelley and Rausch, 2011) or observational studies with time-varying exposure (Barrera-Gomez et al., 2013). However, power tools with a focus on individual differences in change as specified by latent variable models are still lacking. Given recent advances in the formal understanding of statistical power in longitudinal Structural Equation Modeling (e.g., von Oertzen, 2010), the time is ripe to introduce a software tool for the computer-aided design of longitudinal studies. Hence, we propose LIFESPAN, a freely available computer tool for creating linear latent growth curve model (LGCM) designs and for deriving approximate estimates of their statistical power. The currently available version of LIFESPAN allows researchers to explore alternative study designs with equivalent power to detect individual differences in linear change.

In the remainder of this article, we introduce the design principles and specific features of our computational approach, discuss limitations of its current implementation, and lay out a research agenda for the computer-aided design of longitudinal studies.

## Computer-aided design of longitudinal studies: a structural equation modeling approach

Human designers typically envision a design problem in terms of one or more goals they wish to attain, and then consider dimensions that put constraints on the space of admissible solutions, such as cost, time, feasibility, elegance (aesthetics), and ethics. In engineering and the natural sciences, computers often assist humans in finding solutions to design problems of this sort. Computer-aided design (CAD) is devoted to reducing the elapsed time and resources spent during the design task supported by computational facilities (Coons and Mann, 1960). When the goal of a design task is not only feasibility but has further design objectives, the task at hand may be formalized in terms of optimization under constraints (see Rao, 2009). The auspicious role assigned to the computer is to find a solution (e.g., a product) that optimizes one or more criteria under a given set of constraints. In mechanical design, typical goals are the reduction of stress, wear, or weight, for example, minimizing the overall weight in aerospace design or minimizing manufacturing costs in civil engineering design.

Likewise, the planning of a longitudinal study, which involves repeated measurements of one or more variables over time, can be regarded as an engineering task. Generally, researchers have a good sense of their phenomena of interest, and select their measurement instruments on that basis. They then consider various longitudinal study designs based on a collection of reasons that include assumptions about the nature of the change process as well as practical considerations such as available resources (e.g., time and money). This selection process comes with many degrees of freedom, and decisions are often made without full knowledge of their implications. For instance, longitudinal design decisions entail choosing an observational time span, and, within that time span, the frequency and distribution of measurement occasions. Given the complexity and size of the longitudinal design search space, it is surprising that computer-aided approaches to optimal longitudinal design have been largely neglected thus far, despite the longstanding availability of appropriate statistical approaches (e.g., Schlesselman, 1973).

Structural Equation Modeling (SEM; e.g., Bollen, 1989) is a statistical framework that formalizes the relationship between observed and latent variables. SEM notation includes diagrams that represent the entire set of equations underlying a given model (see McArdle and Nesselroade, 2014, pp. 59–66). This feature greatly facilitates the creation, modification, and communication of models, and is particularly useful for comparing different research designs (von Oertzen et al., 2015). Within SEM, latent growth curve models (LGCM) are widely used to capture change in longitudinal data on human behavioral development (e.g., Meredith and Tisak, 1990; Muthén and Curran, 1997; Ferrer and McArdle, 2003, 2010; Duncan et al., 2013). In LGCM, factor loadings represent hypothesized trends over time, such as initial level and linear change. The mean vector, μ, and the covariance matrix, Σ, of the observed variables are a function of factor loadings, Λ, variables'; intercepts, ν, a latent covariance matrix, Ψ, and a residual covariance matrix, Θ (e.g., Bollen, 1989):

$$\begin{array}{l} \Sigma =\Lambda \Psi \Lambda^{\prime }+\Theta \\ \mu =\Lambda \nu \end{array}$$

Under the assumption of homoscedastic and uncorrelated residual errors, the matrices for a linear LGCM are:

$$\begin{array}{l} \;\Lambda =\left[\begin{array}{cc} 1 & t_{1}\\ 1 & t_{2}\\ 1 & \vdots \\ 1 & t_{M} \end{array}\right]\\ \Psi =\left[\begin{array}{cc} \sigma_{I}^{2} & \sigma_{IS}\\ \sigma_{IS} & \sigma_{\text{S}}^{2} \end{array}\right]\\ \;\nu =\left[\begin{array}{c} \mu_{I}\\ \mu_{S} \end{array}\right]\\ \Theta =\left[\begin{array}{ccc} \sigma_{\varepsilon }^{2} & 0 & 0\\ 0 & \ddots & 0\\ 0 & 0 & \sigma_{\varepsilon }^{2} \end{array}\right] \end{array}$$

The parameters in the model are the number of measurement occasions, *M*, at times *t*_1_ to *t_M_*, the residual error, σ^2^_ε_, the mean, μ_*I*_, and variance, σ^2^_*I*_, of the latent intercept, and the mean, μ_*S*_, and the variance, σ^2^_*S*_, of the latent slope, and the latent intercept-slope covariance, σ*_IS_*.

When planning a longitudinal study, statistical consultants are typically approached with questions about the size of the sample needed to approach a level of statistical power that is deemed adequate (e.g., 80%). Questions of this kind have been the target of a large number of simulation studies (e.g., Muthén and Muthén, 2002; Maxwell et al., 2008), which in turn have informed researchers about reasonable ranges for selected designs and effect sizes. So far, however, the curse of dimensionality has rendered an exhaustive simulation-based treatment of statistical power for all potential combinations of design parameters intractable. This impasse can be overcome by statistical theories that formalize parameter trade-off relations in SEM (MacCallum et al., 2010; von Oertzen, 2010).

Specifically, von Oertzen and Brandmaier (2013) have proposed a formal approach, based on power equivalence theory (von Oertzen, 2010), that allows researchers to examine trade-off relations among design parameters of a LGCM while holding statistical power constant. In the context of SEM, power equivalence theory allows the generation of alternative models with different design parameters but equal power according to likelihood-ratio tests. Von Oertzen and Brandmaier (2013) show how power-equivalent operations can be used to transform a given LGCM into alternative models. For tests of interindividual differences of change, σ^2^_*S*_, they present an empirical example of trade-off relations between total study time and the number of measurement occasions. Of course, many more such trade-offs are possible. If multiple measurement instruments were available, combinations of them could be used in multiple-indicator LGCM to increase power (von Oertzen et al., 2010), or the number of participants could be traded for additional bursts or waves of measurement (Schlesselman, 1973; see Raudenbush and Liu, 2001; von Oertzen and Brandmaier, 2013). As is true for any engineering task, the optimal choice among models will depend on external criteria, such as the amount of study time elapsing before targeted effects are reliable, the strain exerted on research participants, and resource expenditures like laboratory space or money.

## Power equivalence theory and effective error

Comparing alternative study designs under equal power allows the optimization of a study design with respect to a given design objective, for example, the minimization of the total study time or the number of measurement occasions or waves. To permit the manipulation of design parameters of a given study design without changing statistical power, we rely on power equivalence theory as introduced by von Oertzen (2010). Two study designs measuring the same effect of interest are power-equivalent if they exhibit the same statistical power to detect the effect. Translating this definition to study designs targeting interindividual differences in change, two study designs are power-equivalent if they have the same power to detect non-zero slope variance in a likelihood-ratio test. Two such study designs may differ in any aspect that does not change the variables involved in the statistical hypothesis. In the context of a test with one degree of freedom (1-*df*), any parameter other than the linear slope may be changed. For example, two alternative study designs may have the same power while differing in a combination of parameters, such as the number of measurement occasions (and thus in the number of observed variables), in the total study time, distribution of measurement occasions in time, precision of the measurement instrument, or the number of participants.

Von Oertzen (2010) noted that together with a given statistical hypothesis, a given, potentially complex SEM can be reduced to a minimal power-equivalent model. For hypotheses about a single latent variable, as in a 1-*df* test of slope variance, power equivalence theory allows the reduction of a structurally complex study design to a simple model with a single *effective error*. This effective error may be interpreted as the hypothetical measurement error encountered had it been possible to measure the latent construct of interest directly. It follows that two alternative study designs with the same effective error are power-equivalent. Thus, the effective error acts as a pivotal point allowing the derivation of power-equivalent models from an initial design. Von Oertzen and Brandmaier (2013) have elaborated this approach for LGCM and hypotheses about the intercept and slope variance. In the following, we reiterate how the effective error in a linear LGCM can be used to arrive at alternative designs given an initial study design.

The effective error of measuring slope variance in a linear LGCM can be written as follows (adapted from Equation 2 in von Oertzen and Brandmaier, 2013):

$$\sigma_{eff}^{2}=\frac{\sigma_{\varepsilon }^{2}}{\sum_{j=1}^{M}t_{j}^{2}-\frac{1}{M+\sigma_{\varepsilon }^{2}/\sigma_{I}^{2}}{\left(\sum_{j=1}^{M}t_{j}\right)}^{2}}$$

where *M* is the number of measurement occasions at time points *t_j_*, σ^2^_ε_ the residual error, and σ^2^_*I*_ the intercept variance. Assuming equally spaced measurements and linear growth over time with *T* being the total study time, *T* = *t_M_*, we can substitute the sums by the following terms:

$$\begin{array}{l} \sum_{j=1}^{M}t_{j}=\sum_{j=1}^{M}\left(\frac{j}{M}T\right)=\frac{1}{2}\left(M+1\right)T\\ \sum_{j=1}^{M}t_{j}^{2}=\sum_{j=1}^{M}{\left(\frac{j}{M}T\right)}^{2}=\frac{\left(M+1\right)\left(2M+1\right)T^{2}}{6M} \end{array}$$

It follows that the effective error is a function of a given set of parameters θ = (σ^2^_ε_, σ^2^_*I*_, *T*, *M*) including residual variance, intercept variance, total study time and number of measurement occasions. Let θ represent the specification of the initial study design. Then, we can define an alternative study design by a second parameter vector $\theta^{\prime }=\left({\sigma^{\prime }}_{\varepsilon }^{2},{\sigma^{\prime }}_{I}^{2},T^{\prime },M^{\prime }\right)$. Both study designs are power equivalent if their effective errors are equal, that is, σ^2^_*eff*_(θ) = σ^2^_*eff*_(θ′). To guarantee power equivalence during manipulation of an alternative design, we allow all but a single parameter in θ′ to be freely varied. Henceforth, we refer to this excluded parameter as *computer-adjusted*. Whenever any value on one of the dimensions of θ′ is changed during the design process, an optimization algorithm is used to adapt the computer-adjusted dimension of θ′ such that σ^2^_*eff*_(θ) = σ^2^_*eff*_(θ′). To accomplish this end we employ a gradient descent algorithm (e.g., Luenberger, 1973) to find the root of σ^2^_*eff*_(θ) − σ^2^_*eff*_(θ′). This general-purpose optimization technique allows us to arrive at alternative models under equivalent statistical power without the need to run computationally expensive Monte Carlo simulations at each optimization step. Sample size can also be a modifiable parameter under power equivalence when the optimization scheme is augmented by numerical approximations of statistical power (see Satorra and Saris, 1985). The layout of a path diagram for an automatically created, alternative study design given by the parameter vector θ′ can either be implemented for a particular design or generally left to an automatic layout algorithm (e.g., Boker et al., 2002).

Based on the design parameters of a LGCM, various indices of design quality other than statistical power itself can be derived. By normalizing the absolute effect size, σ^2^_*S*_, with the effective error, σ^2^_*eff*_, we obtain an index of reliability of the specific likelihood-ratio test of slope variance, effective curve reliability (ECR), which can be interpreted as an effect size estimate of slope variance:

$$ECR=\frac{\sigma_{S}^{2}}{\sigma_{S}^{2}+\sigma_{eff}^{2}}$$

Similarly, growth rate reliability (GRR), introduced by Willett (1989), was used by Rast and Hofer (2014) as an index of statistical power in LGCM. GRR can be regarded as a special case of ECR, in the sense that the two indices yield identical results when the effect of intercept variance on effective error is asymptotically large so that the denominator of the effective error simplifies to a term proportional to the variance of the occasions of measurements. GRR may be more appropriate than ECR if the statistical test used to detect slope variance does not account for the effect of intercept variance (e.g., a one-dimensional Wald test).

In contrast to both ECR and GRR, growth curve reliability (GCR; e.g., McArdle and Epstein, 1987) is a measure of variance explained in the observed variables, and reduces to a scaling of intercept variance and residual variance at the point in time when the regression of the observed variable on the latent slope is zero (i.e., at occasion *j* for which *t_j_* = 0):

$$GCR_{0}=\frac{\sigma_{I}^{2}}{\sigma_{I}^{2}+\sigma_{\varepsilon }^{2}}$$

The different indices serve complementary functions in planning, selecting, and communicating a study. Effective error is particularly useful as a proxy for statistical power when researchers have no clear expectations about the corresponding true score, such as the true variance of change. ECR relates a true score to its effective error, and serves as a proxy for statistical power when sample size and alpha level are left undetermined.

## The LIFESPAN tool

Based on power equivalence theory (von Oertzen, 2010; von Oertzen and Brandmaier, 2013), we have designed LIFESPAN to aid researchers in the design phase of longitudinal studies. The program is freely available at http://www.brandmaier.de/lifespan. With LIFESPAN, our primary goal is to help researchers to design, manipulate, and optimize their longitudinal study design. To this end, researchers using LIFESPAN can: (a) generate a graphical rendition of the model implied by an initial study design; (b) freely and systematically explore the space of alternative power-equivalent study designs; (c) compute and display relevant design indices, such as effective error, GCR, GRR, or ECR; (d) run a Monte Carlo simulation engine to estimate statistical power for a given sample size; and (e) convert the final model from a planning into a data analysis tool. To facilitate this transition, LIFESPAN is based on Ω nyx (von Oertzen et al., 2015), a SEM software environment that is also freely available (http://onyx.brandmaier.de), but distributed as a stand-alone program. LIFESPAN is written in JAVA and runs on all major operating systems, including Linux/Unix, OSX, and Windows. To streamline researchers'; workflow and increase accessibility, we are considering integrating LIFESPAN directly into Ω nyx as a module such that users need not switch between programs when planning a study, running Monte Carlo simulations, and conducting data analyses.

Currently, LIFESPAN is limited to linear LGCM, and is geared toward evaluating the power to detect variance in linear change. Further specification modes, design indicators, and simulation tools related to other design parameters will be added to future releases of the program (see below).

**Figure 1:**
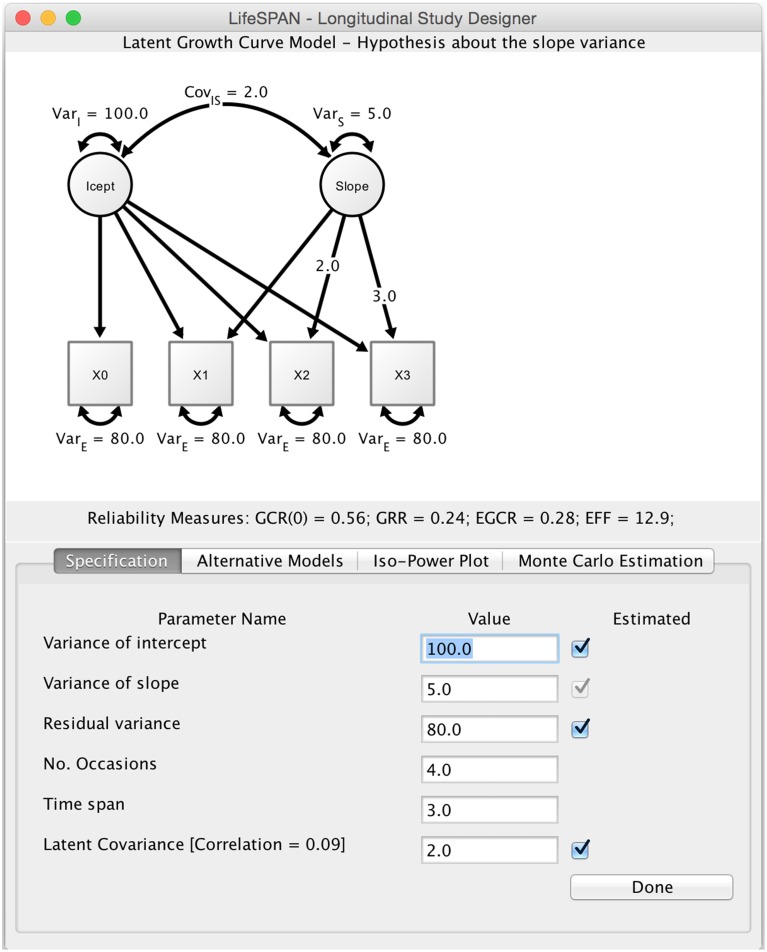
**Main screen of LIFESPAN**. This screenshot shows the specification mode of LIFESPAN. Text fields allow researchers to type in study design parameters, for instance, time span or the number of measurement occasions, and best guesses about true variances in intercept and linear change. At the top, the current study design is displayed as a path diagram.

The main screen of LIFESPAN features three elements (see Figure 1). The top half of the screen displays the path diagram of the initial or target study design. The center shows a summary with a set of study design indices, such as the effective error, GCR, GRR, and ECR. The bottom half of the screen features a control panel. LIFESPAN offers four modes of operation, each corresponding to one of the tabs in the control panel: (1) model specification; (2) alternative models; (3) iso-power plots; and (4) Monte Carlo simulation. In the following, each of these modes is described in detail.

### Model specification

In model specification mode, researchers can specify an initial study design in the form of a linear LGCM. Specification does not require knowledge of syntax or algebra, since researchers are asked to directly manipulate the design parameters of the LGCM. These parameters include the number of measurement occasions, the total time span of the study, and the residual variance of the indicator. In addition, parameters referring to population values at the latent level need to be specified, that is, intercept variance, slope variance, and intercept-slope covariance. Once model specification is completed, clicking *Done* generates a path diagram that corresponds to the specified study design, and delivers the design indices GCR, GRR, ECR, and effective error.

### Alternative models

Proceeding from model specification, LIFESPAN allows researchers to generate alternative models that have equal statistical power to detect variance in linear change. To this end, the parameters chosen during model specification are represented as sliders. In this manner, researchers can observe how different parameter combinations result in identical statistical power.

Specifically, choice buttons allow the selection of one design parameter to be computer-adjusted while the remaining parameters remain user-modifiable. Whenever any of the user-modifiable parameters is changed, the optimization algorithm described above adapts the computer-adjusted parameter such that the resulting alternative study design is power-equivalent to the initial design. As the researcher explores alternative designs, the corresponding path diagram and associated design indices are updated.

### Plots of iso-power contours

To attain a more complete understanding of parameter trade-off relations, the next mode of operation allows researchers to plot iso-power curves (MacCallum et al., 2010; von Oertzen and Brandmaier, 2013). Iso-power curves display power-equivalent alternative models in two-dimensional parameter space; they display bivariate associations between parameters while statistical power to detect linear variance of change is held constant. This feature allows researchers to identify parameter constellations that optimize one or more external criteria, such as total study time and indicator reliability.

### Monte carlo simulation

Finally, LIFESPAN estimates the statistical power to detect variance in linear change for a given sample size. To this end, researchers can choose between two tests: (i) a 1-*df* test of the slope variance; (ii) a generalized variance-covariance likelihood-ratio test with 2-*df* (for discussion, see Hertzog et al., 2008). The current version of LIFESPAN uses a Monte Carlo simulation approach (e.g., Muthén and Muthén, 2002) to estimate actual statistical power. Researchers can specify the sample size and the number of Monte Carlo replications. In each replication, data are simulated from the currently specified study design and are fitted to the same model, once without restriction and once under the restrictions imposed by the selected variance test. Parameter estimation is performed by the estimation engine of Ω nyx (for details, see von Oertzen et al., 2015). By counting the resulting significant likelihood-ratio tests, one obtains an unbiased approximation to the statistical power of the study design.

### Workflow

In its current form, LIFESPAN allows the specification of a longitudinal study design with repeated measures over time in the form of a LGCM. Researchers can enter their initial model parameters and a best guess of the true variance in linear change (e.g., effect size) to obtain approximations to the statistical power to detect between-person differences in linear change. By using sliders that represent various design parameters, researchers can intuitively explore alternative models. Plotting associations between pairs of selected parameters under equal power makes it possible to visualize critical design aspects based on power equivalence theory.

The final model specification can be exported to and directly used in Ω nyx. Ω nyx allows use of the selected design option for Maximum Likelihood estimation of parameters once empirical data have been collected. Also, the graphical interface of Ω nyx allows researchers to expand the model beyond the limitations of LIFESPAN, for instance, by imposing constraints or expanding the model beyond the unconditional LGCM. Further capabilities of Ω nyx include the generation of publication-ready figures, further simulation, and export of the syntax of the final model to three freely available R packages, OpenMx (Boker et al., 2011), lavaan (Rosseel, 2012), and sem (Fox, 2006), and to the commercially available software package M*plus* (Muthén and Muthén, 2007).

## A sample application of lifespan

For illustration, we have recreated a study design taken from the study, “Origins of Variance in the Oldest-Old: Octogenarian Twins” (see Johansson et al., 1999, 2004). Following the values reported by Rast and Hofer (2014; Table 5, line 1, p. 11) for the measure, *Memory-in-Reality Free Recall*, we specified an initial study design with slope variance σ^2^_*S*_ = 0.53, intercept variance σ^2^_*I*_ = 39.63, residual error σ^2^_ε_ = 9.20, intercept-slope covariance σ_*IS*_ = −0.69 (corresponding to an intercept-slope correlation of −0.15), and three measurement occasions spanning a total of 4 years, that is, *T* = 4, and *M* = 3. As reliability and effect size indicators, we obtain GCR of.81, GRR of.32, ECR of.36, and an effective error of.96. Using the Monte Carlo estimation functionality, we estimate the power of the design with a sample size of *N* = 250 to be close to 80%.

**Figure 2:**
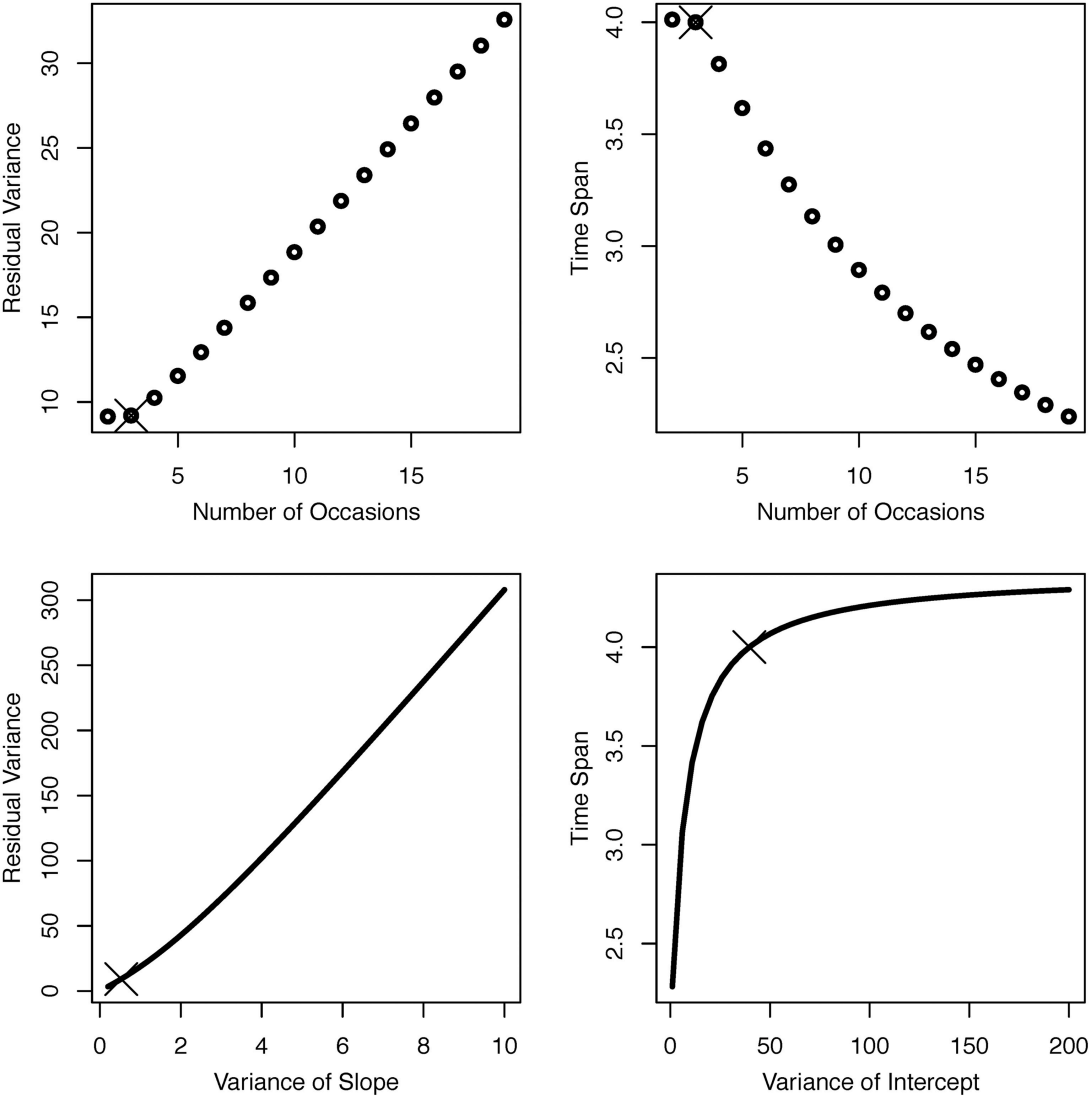
**Iso-power plots for bivariate trade-offs between parameters in a LGCM based on the OCTO-Twin Study**. Number of occasions and residual variance (**top left**), number of occasions and time span (**top right**), variance of slope and residual variance (**bottom left**), and variance of intercept and time span (**bottom right**). The original study design is marked with a cross in each panel.

Based on this empirically realized study design and its observed statistical parameters, we ask four questions concerning possible modifications of the initial design (see Figure 2): (1) If we added (or subtracted) measurement occasions, in how far could we afford to use a less reliable (or would we need a more reliable) measurement instrument? (2) Again, if we added (or subtracted) measurement occasions, by how much could we reduce (or would we need to increase) total study time? (3) If the true variance in linear change was larger (or smaller) than observed, in how far can could we afford a less reliable (or would we need a more reliable) measurement instrument? (4) If individual differences at baseline were higher (or lower) than observed, by how much would we need to extend (or could we reduce) total study time to achieve the same power to detect between-person differences in linear change? The four panels of Figure 2 show iso-power curves that provide answers to each of these questions. Residual variance trades off almost linearly against the number of occasions and the variance of slope (left panels). The number of measurement occasions and total study time span trade off against each other in a quadratic relationship, in the sense that the effect of adding occasions on power is reduced with each additional measurement occasion (upper right panel; cf. von Oertzen and Brandmaier, 2013). Finally, the effect of intercept variance on power quickly reaches an asymptote such that increasing intercept variance needs to be compensated for by only small increments of total study time span to achieve equal statistical power (lower right panel).

## Discussion

### Current limitations of LIFESPAN

We see LIFESPAN as a computational tool that helps researchers to gain insights into trade-off relations among design parameters, and hence enables them to make better decisions about the design of a planned longitudinal study. At the same time, we acknowledge that the current version has at least three important limitations.

First, LIFESPAN is currently limited to linear LGCM. We decided to formalize longitudinal study design in terms of a LGCM because models of this type are widely used for longitudinal data analysis, particularly in lifespan research (Hertzog, 1996; McArdle and Nesselroade, 2003; Lindenberger et al., 2011). We emphasize that the assumption of homogeneous linear change is strong, and quite likely to be incorrect in many empirical settings. For instance, in studies of cognitive aging, changes often accelerate with advancing age (cf. Ghisletta et al., submitted). Hence, we recommend some caution when searching for alternative models, as the linearity assumption may entail substantial misspecification at higher ages, especially when the model covers a large age range.

Second, the current version of LIFESPAN has an exclusive focus on the statistical power to detect between-person differences in linear change. In our judgment, this focus is well justified because the description, explanation, and modification of individual differences in change is central to lifespan theory (Baltes et al., 1977), and arguably the most important reason for conducting longitudinal work in the first place. Accordingly, the indices currently provided by LIFESPAN reflect our substantive research interest in between-person differences in change (Hertzog, 2008; Lindenberger, 2014) and complement our earlier work on statistical power (Hertzog et al., 2006, 2008; von Oertzen et al., 2010; von Oertzen and Brandmaier, 2013).

Third, in the present version of LIFESPAN, power equivalence is based on the 1-*df* test, which refers to the specific test of zero variance. Note that the hypothesis tested in this way is that there is no *unique* variance in linear slope. If the intercept-slope covariance is different from zero, then testing this hypothesis is different from testing the hypothesis of *total* zero variance. When confusing these two hypotheses, manipulating the covariance may yield unintuitive results. To reject the hypothesis of no slope variance in the presence of a non-zero intercept-slope covariance, it is necessary to use the 2-*df* test, or the generalized variance test. It draws power from both the intercept-slope covariance and the slope variance, which also makes it more powerful than the specific variance test (Hertzog et al., 2008; Ke and Wang, 2014). We are currently working on a derivation of the effective error corresponding to this two-dimensional null hypothesis (Brandmaier et al., in preparation) and will implement this derivation in a future version of LIFESPAN. Facilities for the Monte Carlo simulation of statistical power are already available for both the specific and the generalized test of slope variance.

### LIFESPAN as a vehicle for progress in longitudinal study design

The LGCM is just one class of models for evaluating change. It does not directly address the issue of capturing various forms of causality (see Pearl, 2012). Future developments can consider the power to detect fixed and random regression coefficients in alternative structural regression models such as the bivariate dual-change score model (McArdle and Hamagami, 2001; Ferrer and McArdle, 2003; Prindle and McArdle, 2012) as well as continuous time models (Voelkle et al., 2012).

LIFESPAN can be augmented in a number of ways that will enhance its usefulness as a tool to select and evaluate longitudinal study designs. The hope is that we can make LIFESPAN sufficiently flexible to serve as an instrument for promoting progress in longitudinal study design. From this perspective, the current emphasis on linear change as specified in a LGCM is a conservative design limitation that future versions of the program need to overcome. Power equivalence theory, in general, and the notion of effective error, in particular, will play a central role in this endeavor, as the concept of effective error is not limited to testing hypotheses about true variance in change, but can be extended to other effects of a given statistical model. Von Oertzen and Brandmaier (2013) derived an effective error term to detect intercept variance in the context of a LGCM.

In particular, we envision that future versions of LIFESPAN will ultimately include options to specify *variable spacing of measurement occasions* (Willett, 1989; Sliwinski et al., 2010), *selective attrition* (Lindenberger et al., 2002), *cohort-sequential designs* (Schaie, 1965; Baltes, 1968), *non-linear change* (Ghisletta et al., submitted), and *planned missingness* (e.g., McArdle, 1994; Graham et al., 2001; Little et al., 2013; Rhemtulla et al., 2014). Some of these options are discussed in more detail below.

Alternative approaches to sampling time (i.e., occasions of measurement) are important because the density and distribution of measurement occasions influence the statistical power to detect variance in change (Willett, 1989; von Oertzen and Brandmaier, 2013; Rast and Hofer, 2014). More work is needed to find out which time-sampling schemes are well suited to separating long-term change from forms of within-person variability that operate on shorter timescales (Nesselroade, 1991; Lindenberger and von Oertzen, 2006; Sliwinski et al., 2010). Following the original work by Willett (1989), increasing the variance of measurement intervals by giving up the longstanding habit of equally spaced measurement intervals seems highly commendable. Taken to the extreme, the sampling of time can be regarded as a variable that varies randomly across participants (e.g., Voelkle and Oud, 2013).

Regarding selective attrition, future versions of LIFESPAN or related programs would allow researchers to specify drop-out rates, including selection equations to capture possible effects of non-random attrition (cf. Lawley, 1943; Lindenberger et al., 2002). As a first step in this direction, von Oertzen and Brandmaier (2013) derived power-equivalence relations based on score-independent drop-out to examine how power contributions shift from total study time to observation density depending on dropout rate.

It would also be useful to evaluate power in sequential sampling designs that incorporate convergence assumptions (Bell, 1953, 1954; McArdle and Hamagami, 2001; Moerbeek, 2011), and to formally explore the potential consequences of misspecification on the statistical power to detect variance in change (e.g., Sliwinski et al., 2010).

We remind readers that the generation of power-equivalent models requires the specification of population parameters. To the extent that these parameters are biased, unreliable, or simply wrong, the set of power-equivalent models derived on the basis of these parameters will be less useful than desired. Of course, this limitation also applies to Monte Carlo simulations, and to any other method for selection and evaluation of study designs. Von Oertzen and Brandmaier (2013) advised researchers to rely on conservative population values to obtain lower bounds on expected statistical power. Alternatively, it might be useful to treat the uncertainty in population values formally (see Kelley and Rausch, 2011; Lai and Kelley, 2011; Gribbin et al., 2013).

### Outlook

Power evaluation programs such as LIFESPAN serve the purpose of helping researchers to craft and select longitudinal designs that have optimal power to detect random effects of change, based on what is currently known about the change process under investigation. The goal of a fully flexible program that enhances longitudinal study design and obeys the principles of computer-aided design is more attainable than ever before, though a number of difficult problems still need to be resolved.

### Conflict of interest statement

The authors declare that the research was conducted in the absence of any commercial or financial relationships that could be construed as a potential conflict of interest.
